# Proteome-Wide Analysis of Heat-Stress in *Pinus radiata* Somatic Embryos Reveals a Combined Response of Sugar Metabolism and Translational Regulation Mechanisms

**DOI:** 10.3389/fpls.2021.631239

**Published:** 2021-04-12

**Authors:** Ander Castander-Olarieta, Cátia Pereira, Itziar A. Montalbán, Vera M. Mendes, Sandra Correia, Sonia Suárez-Álvarez, Bruno Manadas, Jorge Canhoto, Paloma Moncaleán

**Affiliations:** ^1^Department of Forestry Science, NEIKER, Arkaute, Spain; ^2^Center for Functional Ecology, Department of Life Sciences, University of Coimbra, Coimbra, Portugal; ^3^CNC - Center for Neuroscience and Cell Biology, University of Coimbra, Coimbra, Portugal

**Keywords:** carbohydrates, compatible solutes, heat shock proteins, high temperatures, methylation, proteomics, radiata pine, somatic embryogenesis

## Abstract

Somatic embryogenesis is the process by which bipolar structures with no vascular connection with the surrounding tissue are formed from a single or a group of vegetative cells, and in conifers it can be divided into five different steps: initiation, proliferation, maturation, germination and acclimatization. Somatic embryogenesis has long been used as a model to study the mechanisms regulating stress response in plants, and recent research carried out in our laboratory has demonstrated that high temperatures during initial stages of conifer somatic embryogenesis modify subsequent phases of the process, as well as the behavior of the resulting plants *ex vitro*. The development of high-throughput techniques has facilitated the study of the molecular response of plants to numerous stress factors. Proteomics offers a reliable image of the cell status and is known to be extremely susceptible to environmental changes. In this study, the proteome of radiata pine somatic embryos was analyzed by LC-MS after the application of high temperatures during initiation of embryonal masses [(23°C, control; 40°C (4 h); 60°C (5 min)]. At the same time, the content of specific soluble sugars and sugar alcohols was analyzed by HPLC. Results confirmed a significant decrease in the initiation rate of embryonal masses under 40°C treatments (from 44 to 30.5%) and an increasing tendency in the production of somatic embryos (from 121.87 to 170.83 somatic embryos per gram of embryogenic tissue). Besides, heat provoked a long-term readjustment of the protein synthesis machinery: a great number of structural constituents of ribosomes were increased under high temperatures, together with the down-regulation of the enzyme methionine-tRNA ligase. Heat led to higher contents of heat shock proteins and chaperones, transmembrane transport proteins, proteins related with post-transcriptional regulation (ARGONAUTE 1D) and enzymes involved in the synthesis of fatty acids, specific compatible sugars (myo-inositol) and cell-wall carbohydrates. On the other hand, the protein adenosylhomocysteinase and enzymes linked with the glycolytic pathway, nitrogen assimilation and oxidative stress response were found at lower levels.

## Introduction

As sessile organisms, plants are continuously exposed to a great number of external stimuli and fluctuating stress factors, which are becoming progressively more common due to the on-going climate change situation. Derived from the current global warming trend, heat and drought are becoming a growing concern, as they provoke adverse effects on plant growth and development, significantly determining the productivity and viability of both natural ecosystems and planted forests (Allen et al., 2010; Shen et al., 2015).

Somatic embryogenesis (SE) has long been used as a model to study the different physiological, developmental and biochemical mechanisms underpinning stress response in plants, and it is clear that it can also be used as an interesting tool to modulate the behavior of somatic embryo-derived plants, as postulated by several authors (Kvaalen and Johnsen, 2008; García-Mendiguren et al., 2017).

In previous studies we have reported that high temperatures can determine the success of the different stages of SE, provoking negative effects during initiation but increasing the production of somatic embryos during maturation (García-Mendiguren et al., 2016b; Castander-Olarieta et al., 2019). Those treatments can alter the morphology of embryonal masses (EMs) and Se’s in *Pinus radiata* and *Pinus halepensis*, as well as their hormonal and metabolic profiles (Moncaleán et al., 2018; Castander-Olarieta et al., 2019, 2020a, b; Pereira et al., 2020). Besides, the initial culture conditions can have long-lasting effects, modulating the development and drought stress resilience of somatic plants years later (Castander-Olarieta et al., 2020b), presumably by changes in the expression of stress-related genes and epigenetic marks (Castander-Olarieta et al., 2020c). However, very few proteomic studies have been carried out in pine species addressing heat-stress, and, as far as we know, this is the first report carried out along SE applying such high temperatures.

Heat is responsible for alterations in membrane structures, DNA/RNA stability and cytoskeleton dynamics (Hasanuzzaman et al., 2013; Niu and Xiang, 2018); heat disturbs primary metabolic pathways, leading to internal metabolic imbalances, which in turn can cause the accumulation of toxic reactive oxygen species (ROS). Changes in the redox homeostasis can result in protein denaturation and aggregation, altering the activity of essential enzymes (Paciolla et al., 2016).

In this regard, plants have evolved to rapidly detect and respond to all those factors by modifications and readjustments of a complex molecular machinery. The molecules forming this sophisticated network involve a great variety of metabolites, such as hormones, enzymes and other types of proteins (de Diego et al., 2012, 2013a, b, 2015; Taïbi et al., 2015; Escandón et al., 2017). Besides, recent research has demonstrated that plants can store information from stressful conditions and respond in a more efficient way to future environmental constraints (Turgut-Kara et al., 2020).

Proteomics, which provides the missing link between the genome/transcriptome and the metabolome, allows the identification and quantification of stress-tolerance associated proteins and can be applied as a very useful tool to study stress tolerance in plants (Pinheiro et al., 2014). In the same way, the identification of stress-related proteins gives the possibility to use protein markers to improve selection of elite genotypes with high levels of tolerance to stress, conferring fitness advantages in a climate change scenario (Lippert et al., 2005).

Despite the fact that the diversity of proteins present in a cell is considerably smaller than the number of transcripts, this approach presents numerous advantages if compared to transcriptomics. Proteomics gives a more connected understanding of the phenotype because most biological functions in a cell are executed by proteins rather than by mRNAs, and changes in transcriptome are not always correlated with changes in the abundance of the corresponding protein species (Correia et al., 2016). Proteomics provides important information regarding post-transcriptional and post-translational regulation mechanisms and other factors such as mRNA localization and transport, translation rates, transcript and protein stability, and intercellular protein trafficking (Wang et al., 2013; Passamani et al., 2017). Besides, proteomic studies are very useful when working with non-model plant species because protein sequences are more conserved and allow their identification by comparison with orthologous proteins (García-Mendiguren et al., 2016a).

As a result, proteomics has become a necessary and complementary approach in the post-genomic era, and in recent years, high-throughput techniques have facilitated the study of proteomic responses in several plant species to many different abiotic stresses including cold (Yuan et al., 2019), heat (Escandón et al., 2017), drought (Taïbi et al., 2015), salinity (Passamani et al., 2017), or UV light (Pascual et al., 2017). Several studies in different plant species have identified some protein groups related to heat-stress responses, which include antioxidative enzymes, heat shock proteins (HSPs), proteins related to energy and carbohydrate metabolism, redox homeostasis, protein synthesis and degradation, signal transduction, and transcription factors (Escandón et al., 2017; Parankusam et al., 2017). The latest reports have also highlighted the importance of some nuclear proteins such as histones, methyl cycle enzymes or spliceosome elements during thermopriming and epigenetic-driven regulatory mechanisms (Lamelas et al., 2020).

In this work we are interested in corroborating the hypothesis that the application of heat-stress during initiation of SE in radiata pine (*P. radiata* D.Don) could determine the different stages of the SE process, as well as the protein profile of somatic embryos (Se’s) 7 months later. This approach could give us useful information about the molecular mechanism underpinning stress responses during embryo formation, and shed light on how stress tolerance is built and maintained during the embryogenic process.

To this aim, we have applied a short-gel liquid chromatography coupled to mass spectrometry (Short-GeLC-MS/MS) approach in combination with both data dependent acquisition (DDA) and data independent acquisition (DIA) methods, which is, somehow, novel in the plant scenario. Moreover, we have used the sequential window acquisition of all theoretical fragment ion spectra (SWATH) method (Anjo et al., 2015) which, when compared with the classically employed two-dimensional electrophoresis systems (2-DE), substantially reduces the amount of sample handling and lightens the data analysis process without compromising the protein identification efficiency. Furthermore, this strategy enables the detection of challenging insoluble transmembrane proteins and has great potential when working with non-sequenced species (Heringer et al., 2018).

Finally, in order to have a broader perspective and confirm our results, a high-performance liquid chromatography (HPLC) analysis has been carried out to find whether specific changes in the proteome could have resulted in modifications of several metabolic pathways, i.e., sugar metabolism, which is known to have a key role in stress-response mechanisms (de Diego et al., 2013b).

## Materials and Methods

### Plant Material and Heat Stress Treatments

**FIGURE 1:**
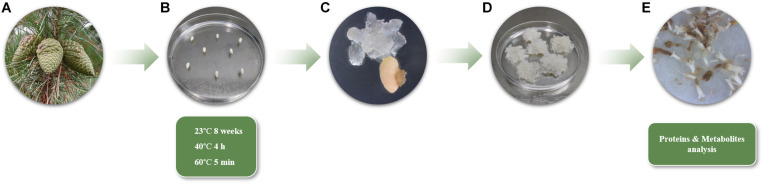
**(A)** Radiata pine green female cones enclosing immature zygotic embryos from five different mother trees. **(B)** Culture of megagametophytes in EDM medium under three different temperature conditions (Cond1 = 23°C, control; Cond2 = 40°C, 4 h; Cond3 = 60°C, 5 min). **(C)** Extrusion of embryogenic tissue from megagametophytes (8 weeks). **(D)** Proliferating EMs (10 weeks). **(E)** Mature Se’s collected for proteins and metabolites analyses (13 weeks).

Green female cones of *Pinus radiata* from five genetically different mother trees in a seed orchard established by Neiker in Deba (Spain, latitude: 43°16′59′′N, longitude: 2°17′59′′W, altitude: 50 m), were collected in June 2018 and processed according to Montalbán et al. (2012) (Figure 1A). Seeds were sterilized following the aforementioned protocol and megagametophytes enclosing immature zygotic embryos were carefully extracted and placed horizontally onto Petri dishes containing Embryo Development Medium (EDM, Walter et al., 2005), supplemented with 3.5 g L^–1^ gellan gum (Gelrite^®^, Duchefa, Haarlem, Netherlands). Intact megagametophytes were subjected to different incubation conditions (Cond1 = 23°C; Cond2 = 40°C, 4 h; Cond3 = 60°C, 5 min) (Figure 1B). In all cases, the Petri dishes were prewarmed to the desirable temperature prior to the cultivation of the megagametophytes. Eight megagametophytes were used per Petri dish, ten Petri dishes per treatment, and 400 megagametophytes were cultured per incubation condition, comprising a total of 1,200 megagametophytes, including all treatments and mother trees. After the treatments, all the samples were kept at 23°C in darkness and the following steps of SE (proliferation and maturation) were carried out at standard conditions as reported in Montalbán and Moncaleán (2018).

After 8 weeks on initiation medium, initiation rates were calculated as the percentage of megagametophytes that had extruded embryonal masses in a sufficient size (7–10 mm of diameter) to be proliferated out of the total number of megagametophytes cultured (Figure 1C). Growing EMs were separated from the megagametophytes and subcultured to a fresh EDM initiation medium. After 14 days, EMs were subcultured onto the same medium but 4.5 gL^–1^ gellan gum every 2 weeks until they reached a sufficient amount of tissue for maturation (2–3 months) (Figure 1D). At that moment, actively growing EMs were recorded as established embryogenic cell lines (ECL), and the percentage of proliferating lines respect to the EMs initiated was calculated following Castander-Olarieta et al. (2019).

Maturation of ECLs was performed as described by Montalbán and Moncaleán (2018), using six replicates (Petri dishes) per ECL and 10 ECLs per treatment. After 13 weeks, maturation success was evaluated, the number of mature and well-formed Se’s per gram of embryogenic tissue was calculated and 100 mg of Se’s (20 to 40 Se’s, depending on their size) per ECL were frozen in liquid nitrogen for both protein and soluble sugar analyses (Figure 1E). Five ECLs were used from 23 and 40°C treatments and 4 ECLs for 60°C treatment, comprising a total of 14 ECLs analyzed.

### Protein and Metabolite Extraction and Protein Mass Spectrometry

Protein and metabolite extractions from mature Se’s were performed following the combined protocol described by Valledor et al. (2014) using 100 mg of liquid nitrogen grinded Se’s. After extraction, metabolites from the polar fraction were saved to new tubes and stored at −80°C until HPLC analysis (explained in section “Soluble Sugar Analysis”) and protein pellets were air dried and re-suspended in 100 μL of solubilisation buffer [7 M urea, 2 M thiourea, 2% (w/v) CHAPS, 1% (w/v) DTT].

As a preparatory step prior to LC-MS analysis, protein extracts (50 μL) were re-precipitated with 4 volumes of cold acetone (250 μL) for 30 min at −80°C. Samples were centrifuged at 20,000 *g*, refrigerated at 4 °C for about 20 min and the pellet was re-suspended in 50 μL of 1 × Laemmli Sample Buffer. The total protein concentration was measured for each sample using the Pierce 660 nm Protein Assay kit (Thermo Scientific^TM^, Waltham, MA, United States). For DDA experiments, samples from each condition (Cond1 = 23°C, control; Cond2 = 40°C, 4 h; Cond3 = 60°C, 5 min) were pooled to create the library of each condition before sample processing and for DIA each sample was processed individually for quantification purposes, as described by Anjo et al. (2015). Protein content from each sample (adjusted based on the protein quantification values obtained previously) was separated by SDS-PAGE for about 17 min at 110 V (Short-GeLC Approach, Anjo et al., 2015) and stained with Coomassie Brilliant Blue G-250. For DDA experiments, each lane was divided into 5 gel pieces (to increase the depth of the analysis and to acquire more fragmentation spectra to correlate with the database) and for DIA experiments into 3 gel pieces for further individual processing. After destaining with a 50 mM ammonium bicarbonate and 30% acetonitrile solution, gel bands were incubated overnight with trypsin for protein digestion and peptides were extracted from the gel using 3 solutions containing different percentages of acetonitrile (30, 50, and 98%) with 1% formic acid. The organic solvent was evaporated using a vacuum-concentrator and peptides were re-suspended in 30 μL of a solution containing 2% acetonitrile and 0.1% formic acid. Each sample was sonicated using a cup-horn (Ultrasonic processor, 750 W) for about 2 min, 40% amplitude, and pulses of 1 s ON/OFF. Ten μL of each sample were analyzed by LC-MS/MS, either for DIA or DDA experiments.

Protein samples were analyzed on a NanoLC^TM^ 425 System coupled to a Triple TOF^TM^ 6600 mass spectrometer (Sciex, Framingham, MA, United States). The ionization source was the OptiFlow^®^ Turbo V Ion Source equipped with the SteadySpray^TM^ Low Micro Electrode (1–10 μL). The chromatographic separation was performed on a Triart C18 Capillary Column 1/32′′ (12 nm, S-3 μm, 150 mm × 0.3 mm, YMC) and using a Triart C18 Capillary Guard Column (0.5 μm × 5 mm, 3 μm, 12 nm, YMC) at 50°C. The flow rate was set to 5 μL min-1 and mobile phases A and B were 5% DMSO plus 0.1% formic acid in water and 5% DMSO plus 0.1% formic acid in acetonitrile, respectively. The LC program was performed as followed: 5–35% of B (0–40 min), 35–90% of B (40–41 min), 90% of B (41–45 min), 90–5% of B (45–46 min), and 5% of B (46–50 min). The ionization source was operated in the positive mode set to an ion spray voltage of 4,500 V, 10 psi for nebulizer gas 1 (GS1), 15 psi for nebulizer gas 2 (GS2), 25 psi for the curtain gas (CUR), and source temperature (TEM) at 100°C. For DDA experiments, the mass spectrometer was set to scanning full spectra (m/z 350-1250) for 250 ms, followed by up to 100 MS/MS scans (m/z 100–1500). Candidate ions with a charge state between +1 and +5 and counts above a minimum threshold of 10 counts per second were isolated for fragmentation and one MS/MS spectrum was collected before adding those ions to the exclusion list for 15 s (mass spectrometer operated by Analyst^®^ TF 1.7, Sciex). The rolling collision was used with a collision energy spread of 5. For SWATH experiments, the mass spectrometer was operated in a looped product ion mode and specifically tuned to a set of 90 overlapping windows, covering the precursor mass range of 350–1,250 m/z. A 50 ms survey scan (350–1,250 m/z) was acquired at the beginning of each cycle, and SWATH-MS/MS spectra were collected from 100 to 1,800 m/z for 35 ms resulting in a cycle time of 3.2 s.

### Soluble Sugar Analysis

For soluble sugar quantification, 200 μL of metabolite samples obtained in Section “Protein and Metabolite Extraction and Protein Mass Spectrometry” were totally dried on a Speedvac to remove the methanol from the extraction buffer and re-suspended in 100 μL distilled water. Soluble sugar and sugar alcohol analysis was performed by HPLC using an Agilent 1260 Infinity II coupled to refractive index detector (RID) (Agilent Technologies, Santa Clara, CA, United States). A Hi-Plex Ca column (7.7 mm × 300 mm, 8 μm) was used for separation of fructose, glucose, sucrose, mannitol and sorbitol. The mobile phase was pure water and the samples were injected in the column at a flow rate of 0.2 mL min^–1^ at 80°C for 40 min. Sugar concentrations were determined from internal calibration curves constructed with the corresponding commercial standards. The concentrations obtained from the HPLC analysis were conveniently adjusted taking into account the initial concentration step (2 times), and results were expressed as μmol g FW^–1^. The mass spectrometry proteomics data have been deposited to the ProteomeXchange Consortium via the PRIDE (Perez-Riverol et al., 2019) partner repository with the data set identifier PXD022711.

### Data Analysis

#### Ion-Library Construction (DDA Information)

A specific ion-library of the precursor masses and fragment ions was created by combining all files from the DDA experiments in one protein identification search using the ProteinPilot^TM^ software (v5.0, Sciex^®^). The paragon method parameters were the following: searched against the reviewed Viridiplantae database (Swissprot) downloaded on 1st April from UniProtKB^1^ (The UniProt Consortium, 2019), cysteine alkylation by acrylamide, digestion by trypsin, and gel-based ID. An independent False Discovery Rate (FDR) analysis, using the target-decoy approach provided by Protein Pilot^TM^, was used to assess the quality of identifications.

#### Relative Quantification of Proteins (SWATH-MS)

SWATH data processing was performed using SWATH^TM^ processing plug-in for PeakView^TM^ (v2.0.01, Sciex^®^). Protein relative quantification was performed in all samples using the information from the protein identification search. Quantification results were obtained for peptides with less than 1% of FDR and by the sum of up to 5 fragments/peptide. Each peptide was normalized for the total sum of areas for the respective sample. Protein relative quantities were obtained by the sum of the normalized values for up to 15 peptides/protein. A correlation analysis between samples was performed using the Spearman Rank Correlation method and considering the relative quantification values determined for all the samples to assure that those from the same condition showed the same behavior.

### Statistical Analysis

The effect of each treatment on the initiation and proliferation rates was evaluated performing a logistic regression and the corresponding analysis of deviance. The mother tree was introduced into the model as a block variable to reduce variability and the Tukey’s *post hoc* test (α = 0.05) was used for multiple comparisons. In the case of the number of Se’s per gram of embryogenic tissue, the usual analysis of variance (ANOVA) did not fulfill the normality hypothesis, and thus, a linear mixed effects model was considered, including the ECL as a random effect with different variance parameters for each treatment level to correct for heteroscedasticity.

For the analysis of the protein results, two different approaches were employed, combining multivariate and univariate analyses. First, we performed a partial least square-discriminant analysis (PLS-DA) using the MetaboAnalyst web-based platform^2^ (Pang et al., 2020) to find out the separation between the three conditions and simultaneously identify the most significant top protein features able to classify the three groups based on variable influence on projections (VIP) values. Those proteins were then clustered based on their biological function according to the FunRich software and the Plants database from UniProtdatabase^3^. In parallel, as cross-validation, a Kruskal–Wallis test was performed to select the proteins which were statistically different between the 3 conditions. The Dunn’s test of Multiple Comparisons, with Benjamini–Hochberg *p*-value adjustment, was performed to determine in which comparisons statistical differences were observed. Finally, proteins overlapping between the Kruskal–Wallis test significant proteins and PLS-DA VIP list were selected and a cluster analysis was performed to them using the MetaboAnalyst web-based platform in order to investigate their relation and relative abundance by generating heatmap and correlation matrix plots. For the heatmap hierarchical clustering the Euclidean distance and the Complete algorithm were used and for the correlation matrix the Pearson’s correlation test was applied.

To assess the effect of the treatments on the levels of each sugar, an ANOVA was conducted followed by multiple comparisons based on Tukey’s *post hoc* test (α = 0.05). When the ANOVA did not fulfill the normality hypothesis the Kruskal–Wallis test was performed.

## Results

### Effect of Temperature Treatments on Somatic Embryogenesis

Analyzing the effect of each treatment on the different steps of SE (initiation, proliferation, and maturation), statistically significant differences were only observed during initiation (*p* < 0.05). Initiation rates were significantly lower at 40°C for 4 h, whereas control and 60°C for 5 min treatments presented similar values (Table 1). Regarding proliferation, no differences could be observed among treatments. The three treatments showed similar proliferation rates, being the ones obtained at 60°C for 5 min slightly higher than the other two (Table 1). Maturation rates were beyond 90% for the three treatments, so temperature did not reduce the maturation capacity of EMs. Although not significant, the number of Se’s produced per gram of embryogenic tissue was higher in samples originating from high temperature culture conditions, especially in those from 40°C for 4 h treatment (Table 1).

**TABLE 1:** Embryonal mass initiation (%) and proliferation rates (%), and number of somatic embryos per gram of embryogenic tissue from *P. radiata* megagametophytes cultured under different temperature conditions.

Treatment	Initiation %	Proliferation %	Se’s g^–1^ ET
23°C, control	44 ± 2.95^a^	31.82 ± 2.09^a^	121.87 ± 45.72^a^
40°C, 4 h	30.5 ± 3.08^b^	30.33 ± 3.01^a^	170.83 ± 53.86^a^
60°C, 5 min	43.5 ± 2.99^a^	36.21 ± 2.54^a^	129.4 ± 41.71^a^

*Data are presented as mean values ± SE. Significant differences at p < 0.05 are indicated by different letters.*

### Relative Quantification of Proteins

Proteomic analysis allowed the identification of 1,020 proteins from the reviewed Viridiplantae database. After data processing 758 proteins were used for quantitative analyses (Supplementary Table 1). The correlation analysis performed demonstrated that samples were highly correlated between all them, with correlation values above 0.72 (data not shown). In the case of samples from the same temperature treatment correlation values were even higher (>0.78).

**FIGURE 2:**
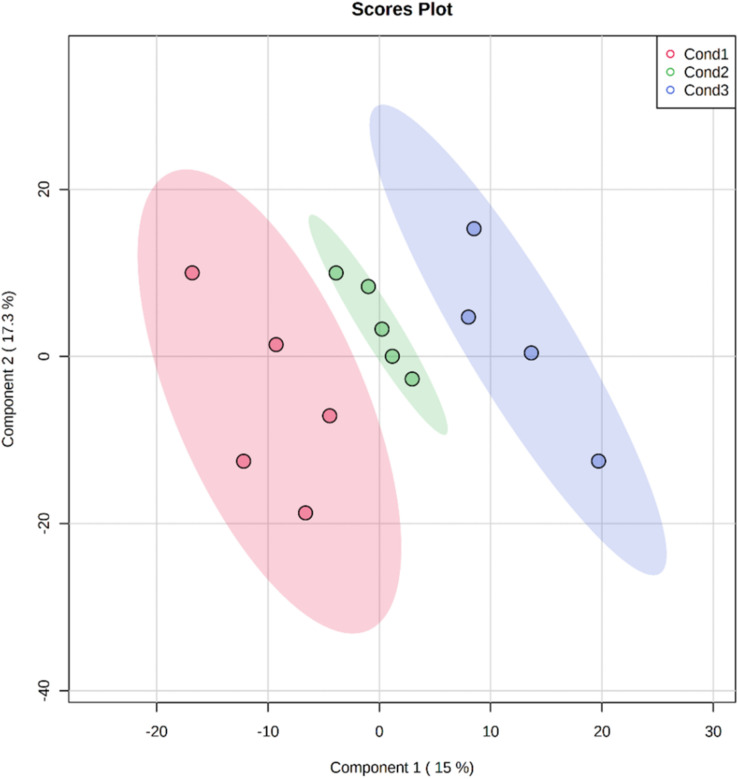
Bi-dimensional representation of the scores for the PLS-DA analysis using the 758 quantified proteins extracted from somatic embryos originating from embryonal masses initiated under three different conditions (Cond1 = 23°C, control: Cond2 = 40°C, 4 h; Cond3 = 60°C, 5 min). Data normalization was performed using the AutoScale method and the scores of the two first components are represented showing the ovals at 95% confidence interval.

For visualization of sample groups and to reduce the complexity of the results, a PLS-DA analysis was performed, a supervised model that uses multivariate techniques to extract via linear combinations of original variables that can predict class membership giving the largest predicted indicator variable. The three conditions employed in this experiment were clearly distinguished and separated by the loading plots of the first and second components of PLS-DA pairwise comparison models (Figure 2). These two components accounted for almost the 33% of the total variance. The first component potentially gathered variability related to heat-stress responses, while the biological function of the second component remained unclear due to an excess of variability.

**FIGURE 3:**
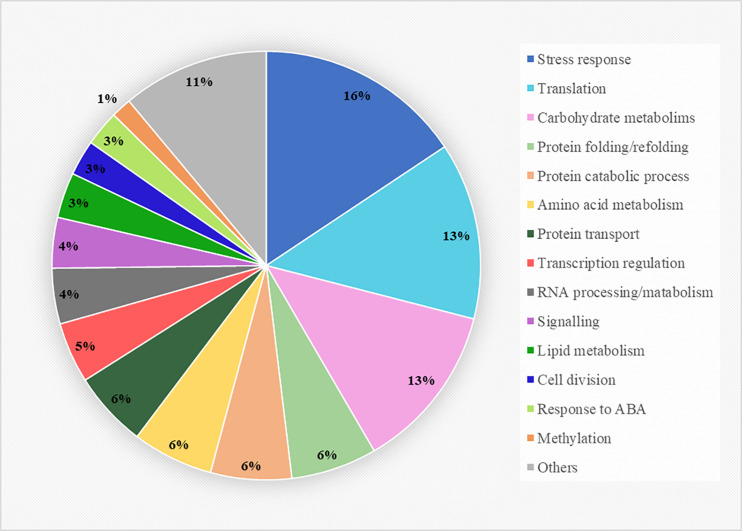
Gene ontology enrichment analysis of the 262 proteins selected from the PLS-DA analysis presenting VIP values greater than 1. The biological function clustering was performed using the FunRich software and the Plants database from UniProtdatabase.

The proteins presenting VIP values greater than 1 were considered the best classifiers, the ones contributing the most to the separation of the three conditions and thus, the ones involved in heat-stress responses. This selection included 262 proteins (Supplementary Figure 1), which were subjected to a gene ontology enrichment analysis to extract information about their mayor biological function. This analysis revealed that the selected proteins belonged to numerous pathways, covering a great number of cellular processes, from primary to secondary metabolism (Figure 3). However, three biological functions were considerably more represented than the other ones, which included proteins involved in direct stress response and adaptation processes, proteins constituents of the translation machinery, translation regulation and proteome reorganization, and proteins involved in carbohydrate metabolism. Other pathways such as amino acid or lipid metabolisms were also represented, although to a lesser extent, and a great proportion of functions related to the life cycle of proteins were detected (folding, catalysis, and transport), accounting for almost the 20% of the total enrichment analysis. At the transcriptome level, some proteins involved in gene expression regulation and RNA processing/metabolism also seemed to be involved in the response to heat, together with signal receptors and proteins constituents of signaling cascades. Finally, a small group of proteins taking part in cell division, response to abscisic acid and methylation processes were also selected by the PLS-DA analysis.

The univariate statistical analysis revealed that 64 proteins were differentially accumulated (*p* < 0.05) between the three conditions (Supplementary Table 2). Comparing these proteins with the 262 proteins selected from the PLS-DA analysis we observed that 54 proteins were common between both statistical approaches and were considered the top significant proteins after heat-stress response in our samples (Supplementary Figure 2). The complete list of these proteins, their accession number, the fold-change between conditions, and the results from both statistical analyses are shown in Tables 2, 3.

**TABLE 2:** Top significant proteins selected from the combination of univariate statistical analysis (Kruskal–Wallis test) and multivariate PLS-DA analysis (*p* < 0.05 and VIP > 1) in somatic embryos of *P. radiata* originating from embryonal masses induced under high temperature conditions (Cond1 = 23°C, control; Cond2 = 40°C, 4 h; Cond3 = 60°C, 5 min).

		Fold change			Kruskal–Wallis	PLS-DA
Description	Accession	Cond2/Cond1	Cond3/Cond1	Cond3/Cond2	*p*-value	VIP
60S ribosomal protein L3-2	P22738	0.84	0.55	0.65	0.005	2.43
Small heat shock protein, chloroplastic	Q95661	2.68	1.94	0.72	0.007	1.38
Methionine–tRNA ligase, cytoplasmic	Q9SVN5	0.35	0.12	0.34	0.009	2.22
Outer plastidial membrane protein porin	P42054	1.1	1.58	1.43	0.01	2.25
Mitochondrial outer membrane protein porin 2	Q6L5I5	0.91	1.38	1.51	0.011	1.9
DnaJ protein homolog	Q04960	1.42	1.51	1.06	0.013	2.17
Unknown protein 3 (Fragment)	P85487	0.66	0.47	0.71	0.013	2.12
40S ribosomal protein S12	Q9XHS0	1.39	1.76	1.27	0.015	2.28
Thiamine biosynthetic bifunctional enzyme BTH1, chloroplastic	O48881	1.03	0.71	0.69	0.016	1.86
Translationally-controlled tumor protein homolog	Q9ZRX0	0.85	0.68	0.8	0.017	2.21
Ketol-acid reductoisomerase, chloroplastic	Q65XK0	0.74	0.4	0.53	0.017	2.22
Ribulose bisphosphate carboxylase/oxygenase activase, chloroplastic	P93431	0.71	0.58	0.82	0.018	2.42
Actin-2	P0C539	1.3	0.4	0.31	0.018	1.51
Probable fructokinase-6, chloroplastic	Q9C524	0.7	0.65	0.93	0.018	2.08
Ferredoxin–nitrite reductase, chloroplastic	Q39161	0.59	0.64	1.09	0.018	1.51
60S ribosomal protein L23	Q9XEK8	0.98	1.37	1.4	0.018	2.11
Adenosylhomocysteinase	P68173	0.93	0.22	0.24	0.018	1.98
Pyruvate dehydrogenase E1 component subunit beta-1	Q6Z1G7	0.89	0.69	0.78	0.02	2.27
Ribulose bisphosphate carboxylase/oxygenase activase A	Q40073	0.68	0.52	0.77	0.02	2.35
Sugar transporter ESL1	Q94KE0	1.19	1.77	1.49	0.021	1.85
Casein kinase II subunit alpha-2	Q9AR27	0.63	0.71	1.13	0.021	1.67
Protein translation factor SUI1 homolog	Q9SM41	1.02	1.23	1.21	0.021	1.96
Probable RNA-binding protein ARP1	Q9M1S3	1.36	1.74	1.28	0.022	2.38
Polyadenylate-binding protein 8	Q9FXA2	1.24	1.57	1.27	0.022	2.37
Aminopeptidase M1	Q8VZH2	0.56	0.5	0.89	0.025	1.97
Phosphomannomutase	Q1W374	0.95	0.65	0.68	0.026	2.23

*Fold changes are presented as the ratio between one condition and the other.*

**TABLE 3:** Continuation of Table 2.

		Fold change			Kruskal–Wallis	PLS-DA
Description	Accession	Cond2/Cond1	Cond3/Cond1	Cond3/Cond2	*p*-value	VIP
Probable UDP-arabinopyranose mutase 1	O04300	1.02	0.54	0.53	0.026	1.9
60S ribosomal protein L30-3	Q9LSA3	1.02	0.76	0.75	0.026	1.8
Inosine triphosphate pyrophosphatase	C5WZH0	0.84	0.8	0.95	0.026	2.11
4-coumarate–CoA ligase-like 5	Q7F1X5	0.38	0.14	0.37	0.026	1.76
60S ribosomal protein L17	O48557	0.94	1.4	1.49	0.027	1.83
Probable sucrose-phosphate synthase 2	O04933	0.96	0.53	0.56	0.027	2.05
40S ribosomal protein S2-1	Q8L8Y0	1.15	1.36	1.18	0.028	2.21
60S ribosomal protein L4-1	Q9SF40	2.93	3.55	1.21	0.03	1.85
Eukaryotic translation initiation factor 3 subunit D	P56820	1.2	1.46	1.22	0.032	1.97
Proteasome subunit alpha type-6	Q9XG77	0.88	0.81	0.91	0.032	1.98
Protein argonaute 1D	Q5Z5B2	1.67	1.66	0.99	0.032	1.48
Threonine synthase, chloroplastic	Q9MT28	0.87	0.77	0.89	0.033	2.1
Probable inositol 3-phosphate synthase isozyme 3	Q9LX12	1.97	3.2	1.62	0.034	2.29
Proteasome subunit alpha type-7-A	Q6YT00	0.81	0.64	0.79	0.034	2.01
Protein EXPORTIN 1A	Q9SMV6	1.09	0.43	0.39	0.035	1.25
Ras-related protein RIC1	P40392	0.59	0.36	0.6	0.037	1.82
V-type proton ATPase subunit E	Q9SWE7	1.02	1.25	1.22	0.038	1.71
3-ketoacyl-CoA thiolase 1, peroxisomal	Q8LF48	0.98	0.72	0.74	0.038	1.87
Importin subunit alpha-2	F4JL11	1.05	0.76	0.72	0.039	1.66
Acetyl-coenzyme A carboxylase carboxyl transferase subunit alpha, chloroplastic	Q41008	1.06	1.46	1.37	0.039	1.83
Enolase	Q43321	0.93	0.04	0.05	0.039	2.1
Probable phospholipid hydroperoxide glutathione peroxidase	O23814	0.69	0.62	0.89	0.04	2.07
Mitochondrial outer membrane protein porin 5	Q84P97	1.09	1.28	1.17	0.042	1.94
DnaJ protein homolog ANJ1	P43644	1.29	1.34	1.04	0.046	1.87
60S ribosomal protein L26-2	Q9FJX2	1.02	1.36	1.33	0.046	1.86
Ribonuclease TUDOR 2	Q9FLT0	0.67	0.69	1.03	0.047	1.76
Cytochrome c oxidase subunit 6b-3	Q9SUD3	1.19	1.47	1.24	0.048	2.04
UDP-glucuronic acid decarboxylase 4	Q8S8T4	1.1	1.31	1.19	0.048	2.02

*Top significant proteins selected from the combination of univariate statistical analysis (Kruskal–Wallis test) and multivariate PLS-DA analysis (p < 0.05 and VIP > 1) in somatic embryos of P. radiata originating from embryonal masses induced under high temperature conditions (Cond1 = 23°C, control; Cond2 = 40°C, 4 h; Cond3 = 60°C, 5 min). Fold changes are presented as the ratio between one condition and the other.*

**FIGURE 4:**
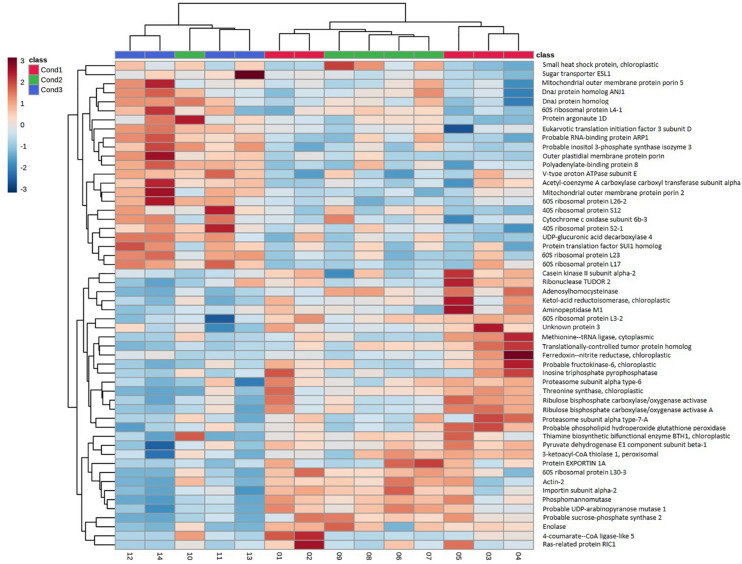
Hierarchical clustering heatmap using the 54 proteins selected from the combination of Kruskal–wallis test and the PLS-DA analysis in somatic embryos of *P. radiata* originating from high temperature conditions (Con1 = 23°C, control; Cond2 = 40°C, 4 h; Cond3 = 60°C, 5 min). Hierarchical clustering was performed at the protein (rows) using Euclidean distance and Complete for the clustering algorithm.

In order to better visualize the relation between proteins, a cluster analysis was performed with the selected 54 top significant proteins by generating a heatmap (Figure 4) and a correlation matrix (Supplementary Figure 3). These analyses showed two clearly differenced groups of proteins based on their relative abundance between conditions. One of the groups presented an abundance increasing tendency from the control condition of 23°C (Cond1) to the highest temperature condition of 60°C (Cond3). The condition of 40°C for 4 h (Cond2) showed an intermediate behavior. As observed in the correlation matrix, all the proteins from this group were closely interrelated, presenting high positive correlation coefficients. This protein group included a great variety of proteins, among which some of them were specific of the group: HSPs and chaperones (small heat shock protein, DnaJ protein homolog ANJ1, DnaJ protein homolog) and proteins involved in ion and small molecules transport (outer plastidial membrane protein porin, mitochondrial outer membrane protein porin 2, mitochondrial outer membrane protein porin 5, sugar transporter ESL1). Some proteins related to the synthesis of specific sugars and sugar alcohols were also over-accumulated under high temperatures, such as UDP-glucuronic acid decarboxylase 4 and inositol 3-phosphate synthase isozyme 3. Following the same pattern, a great number of structural constituents of ribosomes and translation regulation factors were detected (40S ribosomal protein S1, 60S ribosomal protein L23, 60S ribosomal protein L17, 40S ribosomal protein S2-1, 60S ribosomal protein L4-1, 60S ribosomal protein L26-2, protein translation factor SUI1 homolog, polyadenylate-binding protein 8, eukaryotic translation initiation factor 3 subunit D). Finally, it is also remarkable the observed higher amount of proteins involved in the post-transcriptional regulation of RNA metabolism at high temperatures, such as RNA-binding protein ARP1 and protein ARGONAUTE 1D, and of one protein that takes part in the fatty acid biosynthetic process (acetyl-coenzyme A carboxylase carboxyl transferase subunit alpha). Besides, some of these proteins presented high fold changes between control (Cond1) and treatments (Cond2 and Cond3), such as small heat shock protein, 60 S ribosomal protein L4-1 and inositol 3-phosphate synthase isozyme 3 (Tables 2, 3).

On the contrary, the other subgroup of proteins showed reduced abundance levels under high temperature conditions, especially at 60°C for 5 min (Figure 4), and was negatively correlated with the first group of proteins (Supplementary Figure 3). This cluster was composed to a vast extent by central metabolism enzymes, covering diverse pathways such as, glycolysis, citric acid cycle, fatty acid beta-oxidation or Calvin cycle (pyruvate dehydrogenase E1 component subunit beta-1, enolase, ribulose bisphosphate carboxylase/oxygenase activase A, ribulose bisphosphate carboxylase/oxygenase activase, 3-ketoacyl-CoA thiolase 1), and some proteins involved in the synthesis of specific sugars such as fructokinase-6, sucrose-phosphate synthase 2, UDP-arabinopyranose mutase 1 and phosphomannomutase. Proteins involved in oxidative-stress defense were clearly down-expressed (thiamine biosynthetic bifunctional enzyme BTH1, 4-coumarate–CoA ligase-like 5, phospholipid hydroperoxide glutathione peroxidase, EXPORTIN 1A), together with those implicated in amino acid synthesis and protein catalysis (threonine synthase, ketol-acid reductoisomerase, proteasome subunit alpha type-6, proteasome subunit alpha type-7-A, aminopeptidase M1). Other proteins found at lower concentrations at high temperatures included structural constituents of the cytoskeleton (actin-2, translationally-controlled tumor protein homolog), some proteins related with translation (60S ribosomal protein L3-2, 60S ribosomal protein L30-3, and methionine–tRNA ligase), post-transcriptional gene regulation and epigenetic mechanisms (ribonuclease TUDOR 2, adenosylhomocysteinase), nitrogen metabolism (ferredoxin–nitrite reductase), cellular receptors for nuclear import (importin subunit alpha-2) or electron transport proteins (cytochrome c oxidase subunit 6b-3). It is noticeable that some of them presented high fold changes: enolase, methionine–tRNA ligase, adenosylhomocysteinase, and 4-coumarate–CoA ligase-like 5 (Tables 2, 3).

### Soluble Sugar Content Quantification

The HPLC approach revealed the presence of three (fructose, glucose, and sucrose) of the five sugar and sugar alcohols analyzed. Sorbitol and mannitol could not be detected in Se’s using this technique. All sugars presented similar concentrations, although the levels of sucrose and fructose were slightly higher than those of glucose (Table 4). Taking into account the effect of the treatments, no significant differences were observed. However, the levels of sucrose were on the verge of statistical significance (*p* = 0.078), showing a decreasing tendency at high temperatures, especially at 60°C for 5 min treatment (%23 decrease with respect to control treatment of 23°C). This pattern was not appreciable for the rest of sugars detected, although the lowest levels were also observed at 60°C for 5 min treatment (Table 4).

**TABLE 4:** Effect of temperature treatment (23°C, control; 40°C, 4 h; 60°C, 5 min) on the levels of the following sugars and sugar alcohols in somatic embryos of *P. radiata*: fructose, glucose and sucrose (μmol g FW^–1^).

Treatment	Fructose	Glucose	Sucrose
23°C, control	70.88 ± 1.95^a^	46.77 ± 5.03^a^	72.98 ± 5.34^a^
40°C, 4 h	74.57 ± 3.31^a^	46.75 ± 5.64^a^	67.41 ± 5.88^a^
60°C, 5 min	66.12 ± 6.55^a^	40.76 ± 6.26^a^	56.19 ± 3.52^a^

*Data are presented as mean values ± SE. Significant differences among treatments at p < 0.05 are indicated by letters.*

## Discussion

In this work, we have tried to study the proteome of Se’s to obtain insights about the effect of heat-stress at early stages of radiata pine SE and elucidate how that effect can modulate the success of different stages of the process.

Following the same tendency observed in previous studies (Castander-Olarieta et al., 2019), in this work the combination of high temperatures and long exposure time-periods (40°C, 4 h) provoked a considerable decrease of around 13% in the initiation success of EMs. This could be attributed to oxidative damage and structural imbalances at cellular and tissue level, as reported in Castander-Olarieta et al. (2019). On the other hand, no differences were observed at the proliferation stage. Castander-Olarieta et al. (2019) reported that the detrimental effects of the application of high temperatures during initiation could also be observed after four subculture periods (2 months) during proliferation. Results obtained at this stage seem quite ambiguous, as other authors reported higher proliferation rates when EMs were induced under stressful conditions (Fehér, 2015; García-Mendiguren et al., 2016b). This fact could be attributed to the type and dose of the applied stress and the capacity of EMs to overcome the consequences of stress.

Although not significant, the number of Se’s increased under high temperatures, a long-observed trend in our laboratory for both *P. radiata* and *P. halepensis* EMs initiated under high temperatures (García-Mendiguren et al., 2016b; Castander-Olarieta et al., 2019; Pereira et al., 2020). The same pattern was detected in similar temperature-based experiments during initiation of *Pinus pinaster* SE (Arrillaga et al., 2019), confirming the role of temperature as an activator of embryo development. On top of that, Castander-Olarieta et al. (2019) reported altered morphologies in both EMs and Se’s originating from different temperature treatments, and despite not observing germination and acclimatization differences, the resulting somatic plants showed changes at phenotypic level in terms of growth and stress-resilience (Castander-Olarieta et al., 2020a). To shed light on these phenomena, the proteome of Se’s was analyzed.

The proteomic analysis enabled the identification of 1,020 proteins; similar values to the ones obtained at plant level in radiata pine (Escandón et al., 2017) and in somatic embryos of other pine species (Morel et al., 2014b). Multivariate statistical analysis of the proteomic results revealed a large amount of proteins differentially responding to the three temperature conditions even several months later at Se’s stage. This fact could explain the altered behaviors described during the different stages of SE in this study and previous ones. These proteins covered a broad spectrum of biological and molecular functions, but three groups were clearly more represented, including proteins involved in stress responses, proteome readjustment and carbohydrate metabolism. In spite of being at lower levels, proteins taking part in other interesting metabolic pathways were also detected, such as amino acid and lipid synthesis, transcription regulation, RNA metabolism and methylation. These results are in agreement with other studies carried out in radiata pine plantlets subjected to different stress conditions (Escandón et al., 2017; Pascual et al., 2017; Lamelas et al., 2020).

Further statistical analyses combining multivariate with univariate approaches highlighted the importance of 54 proteins as the top significant ones contributing to the separation between the three conditions. Among these proteins, it is noticeable the higher presence of HSPs and molecular chaperones in Se’s originating from EMs induced at high temperatures. HSPs are up-regulated under supra-optimal high temperatures (Yuan et al., 2019; Jiang et al., 2020) and prevent proteins from being denatured by assisting in protein folding and processing. In that way, proteins can maintain a proper stability and function under unfavorable conditions (Hasanuzzaman et al., 2013; Krishnan et al., 2020).

Many studies have observed that these types of molecules are essential for heat-tolerance (Meiri and Breiman, 2009; Perez et al., 2009). In fact, while other stress responsive proteins such as ROS detoxifying enzymes have been described as important mediators in basal thermo-tolerance and early response processes, HSPs and chaperones appear to be required for both basal and acquired thermo-tolerance (Bokszczanin and Fragkostefanakis, 2013), even contributing to plant acclimation (Escandón et al., 2017). Reinforcing this idea, there are several examples of transgenic plants constitutively expressing HSPs that without a previous heat treatment exhibit an enhanced thermo-tolerance (Lee et al., 1995; Malik et al., 1999). As a result, it would be interesting to confirm if this protein profile is maintained throughout the SE process, influencing the *ex vitro* performance of the generated plants, as observed at the physiological level under control and drought stress conditions in Castander-Olarieta et al. (2020b). Besides, the accumulation of HSPs has also been observed under other abiotic stress conditions, including drought (Kosová et al., 2011; Taïbi et al., 2015), which could provide simultaneous protection against more than one environmental constraint.

Chaperones and HSPs seem to be closely related with many other proteins in cells, such as post-transcriptional regulation elements. This is the case of protein ARGONAUTE 1D (Q5Z5B2), which was found at higher concentrations under high temperature conditions, positively correlating with HSPs. Similar responses have also been observed in other plant model systems (Zhang et al., 2012). These types of proteins bind to short RNAs such as microRNAs (miRNAs) or short interfering RNAs (siRNAs), and repress the translation of mRNAs which are complementary to them. These gene expression regulatory mechanisms have been reported to be key players during the formation of temperature-dependent epigenetic memory in Norway spruce (Yakovlev and Fossdal, 2017). However, other proteins related with post-transcriptional regulation, such as the ribonuclease TUDOR 2, were down-regulated under high temperatures, contradicting the studies that remark the interplay between HSPs, stress granules and processing bodies (Miroshnichenko et al., 2005). In this sense, TUDOR 2 is essential for the integrity and function of stress granules and processing bodies, which apart from protecting RNAs from harmful conditions (Jang et al., 2020), seem to be important sites of post-transcriptional gene regulation (Gutierrez-Beltran et al., 2015).

Adenosylhomocysteinase, another protein related to epigenetic regulation, showed a decreasing pattern in Se’s from high temperatures, especially at 60°C for 5 min. It controls methylation through regulation of the intracellular concentration of adenosylhomocysteine, a competitive inhibitor of *S*-adenosyl-L-methionine-dependent methyl transferase reactions. These data suggest that heat-stress might have derived in hypomethylation of Se’s, as observed in embryonal masses and somatic plants originating from the same temperature treatments in previous studies (Castander-Olarieta et al., 2020c). Similar results were obtained by Lamelas et al. (2020), indicating that DNA methylation, together with post-transcriptional regulation mechanisms may be involved in the maintenance of acquired thermotolerance and in the establishment of an epigenetic memory. It would be interesting to perform further analyses to confirm this hypothesis and study its evolution along the whole SE process to see if the altered behavior of the resulting somatic plants in Castander-Olarieta et al. (2020b) could be attributed to this fact.

In the same way, the proteome analysis revealed that the translasome machinery, including ribosomal proteins and translation regulation factors, together with proteins involved in protein catalysis and homeostasis such as different proteasome subunits, had a relevant role during heat-stress response, as already described in other studies (Wang et al., 2018; Li et al., 2019). Although varying patterns were observed under the different temperature conditions, most of the ribosomal proteins and all the translation regulation factors followed an accumulation tendency when heat was applied, while all the proteins involved in protein degradation were down-represented. In fact, one of the proteins presenting the highest fold-changes was the 60S ribosomal protein L4-1, which was detected at higher concentrations at both 40 and 60°C treatments. This protein has been found to be temperature sensitive, increasing considerably its expression under cold (Schlaen et al., 2015). As a result, we can state that heat provokes a deep reorganization of the proteome, by a strict regulation of translation and an altered ribosome composition that could result in a selective mRNA translation to better cope with adverse conditions.

Besides, the relative abundance and differential expression of the aforementioned proteins seem to have further implications in different plant developmental processes via interconnections with phytohormones and other signaling molecules. For example, the function of some ribosomal proteins is linked with cytokinin molecular circuits (Černý et al., 2013). Interestingly, the application of the same high temperature conditions at initiation of *P. radiata* and *P. halepensis* SE provoked changes in the cytokinin profile of EMs (Castander-Olarieta et al., 2020a, b; Pereira et al., 2020). It would be of special interest for future studies to confirm if those changes are maintained in Se’s and study their relationship with the translasome machinery.

Apart from that, protein homeostasis seems to have special relevance during conifer SE, as indicated by the differential expression of specific proteasome subunits and translation elongation factors during EM proliferation and Se’s development in numerous conifer species (Trontin et al., 2016). Specifically, at maturation stage the synthesis of major storage proteins of the globulin and albumin families and proteins involved in acquisition of desiccation tolerance (late embryogenesis abundant proteins) appear to be of great relevance, even determining the germination capacity of Se’s (Klimaszewska et al., 2004; Businge et al., 2013). Furthermore, Lippert et al. (2005) proposed the proteasome complex as a molecular marker of embryo development. Consequently, the different proteasome and translation regulation factors profiles observed in this study could be associated with the enhanced capacity of EMs subjected to heat-stress to produce Se’s and with the different morphologies observed in Castander-Olarieta et al. (2019) among Se’s originating from different temperature treatments.

Heat also induced changes in the metabolism of Se’s. The levels of acetyl-coenzyme A carboxylase carboxyl transferase subunit alpha, an enzyme that takes part in a sub-pathway of the fatty acid biosynthetic process, was clearly accumulated at high temperatures. Heat-stress increases membrane fluidity and as a result plants need to alter fatty acid composition of lipids to compensate for those changes (Escandón et al., 2017).

On the other hand, the glycolytic pathway, as well as the citric acid cycle and β-oxidation of fatty acids appeared to be less active in samples originating from high temperatures, as the proteins pyruvate dehydrogenase E1 component subunit beta-1, enolase and 3-ketoacyl-CoA thiolase 1 were found at lower levels. Parankusam et al. (2017) and Wang et al. (2018) also observed the same pattern when applying heat to chickpea and radish plants, respectively. A decrease in carbon catabolism has also been detected under favorable maturation conditions in *P. pinaster* EMs, leading to high quality embryos (Morel et al., 2014a). However, other reports in *Picea glauca* and some broad-leaf woody plants highlight the importance of high enolase activity as a marker of embryogenic character and normal embryo development (Lippert et al., 2005; Correia et al., 2012).

In the same way, the levels of enzymes involved in the synthesis of starch and sucrose (fructokinase-6 and sucrose-phosphate synthase 2) were reduced under high temperatures, as confirmed by the HPLC analysis for sucrose. Despite this fact, the levels of sucrose were considerably higher in all samples if compared with those from EMs in previous studies (Castander-Olarieta et al., 2019). The increase of sucrose during the formation of Se’s has long been documented as a symbol of acquisition of desiccation tolerance (Businge et al., 2013).

The simultaneous decrease of the glycolytic activity and of the synthesis of disaccharides and polysaccharides would result in the accumulation of free soluble monosaccharides like glucose or fructose. However, this was not observed at the HPLC analysis, and thus, we could assume that these molecules might be serving as precursors for the synthesis of other specific compounds. In fact, inositol 3-phosphate synthase isozyme 3, the key enzyme for the synthesis of myo-inositol from glucose, was notably accumulated under high temperatures, as already observed in other plant species (Khurana et al., 2012). This molecule acts as a compatible solute, maintaining plant cell turgor and stabilizing membranes and proteins (Bokszczanin and Fragkostefanakis, 2013). Nonetheless, the levels of mannitol and sorbitol, molecules previously identified as important osmolytes under heat and drought (Alhaithloul, 2019; Ivanov et al., 2019), were not detectable in radiata pine Se’s using HPLC.

Likewise, the differential expression of phosphomanno- mutase, UDP-arabinopyranose mutase and UDP-glucuronic acid decarboxylase 4 also suggests a drift from glycolysis and starch accumulation toward the synthesis of cell-wall non-cellulosic polysaccharides. Specifically, UDP-glucuronic acid takes part in the synthesis of D-xylose, a constituent component of cell-wall polymers whose concentration has been demonstrated to change under heat-stress (Lima et al., 2013).

In correlation with these enzymes we found a huge amount of proteins involved in oxidative stress response processes and in the synthesis of certain amino acids. All of them showed a decreasing tendency in Se’s originating from high temperature conditions, probably conditioned by the decreased in central metabolism activity, the reduced availability of carbon skeletons and a simultaneous reduction of nitrogen assimilation (lower levels of ferredoxin–nitrite reductase). These proteins included an enzyme responsible of the synthesis of thiamine (thiamine biosynthetic bifunctional enzyme BTH1), which provides oxidative stress resistance via regulation of glucose metabolism (Kartal and Palabiyik, 2019), and 4-coumarate–CoA ligase-like 5, involved in the flavonoid and phenylpropanoid biosynthesis pathway, whose accumulation is essential for the prevention of oxidative damage (Eliášová et al., 2017). Phospholipid hydroperoxide glutathione peroxidase, which protects cells and enzymes from oxidative damage (Yant et al., 2003) was also included in this group. Similar results were obtained for EXPORTIN 1A, a protein involved in heat-induced oxidative stress basal resistance (Wu et al., 2010), and threonine synthase, whose product, threonine, is the precursor of isoleucine (Joshi et al., 2010), other amino acid whose enzyme was down-expressed (ketol-acid reductoisomerase). Previous studies carried out in our laboratory (Castander-Olarieta et al., 2019) reported the accumulation of phenolic compounds and branched chain amino acids (isoleucine) in EMs at first subculture after heat application, in opposition to the results from this study in Se’s months later. As a result, we can assume that the response to oxidative stress is more relevant during short-term heat exposures, as observed by Escandón et al. (2017), rather than during acclimatization or acquired tolerance.

Finally, it is noticeable that several transmembrane transport proteins (porins) and sugar transporters were found at higher levels under high temperatures. Several authors have remarked the importance of these proteins during osmotic stress adaptation and thermal sensing (Brumós et al., 2009; Yakovlev et al., 2016). Based on the positive correlation of these proteins and the increased levels of specific compatible solutes such as myo-inositol, we can hypothesize that a synergistic response is taking place for an enhanced and controlled transport of osmolytes in Se’s originating from heat stress conditions.

This study provides novel information about the long-term effect of heat-stress on radiata pine SE at the proteome level. Results indicated a complex and selective reorganization of the proteome, combined with the possible activation of certain epigenetic and post-transcriptional regulation mechanisms, as well as the accumulation of HSPs and chaperones and a drift in the carbohydrate metabolism toward the synthesis of fatty acids, specific compatible solutes and cell-wall remodeling carbohydrates. These results suggest that the application of stress during initial steps of the SE process could provoke delayed effects in embryogenic cells leading to changes in the subsequent stages up to plant level. This has great potential as the starting point of further research and toward practical applications such as modulating the characteristics of plants using somatic embryogenesis.

## Data Availability Statement

The data has been uploaded to the PRIDE repository with the dataset identifier PXD022711.

## Author Contributions

PM, IM, JC, SC, CP, and AC-O conceived and planned the experiments. AC-O prepared all the plant material and executed the heat stress experiments. AC-O, CP, VM, and SC carried out the protein analysis. AC-O, CP, and SS-Á performed the soluble sugar content quantification. AC-O, VM, SC, and BM carried out the data curation and statistical analysis. AC-O and VM wrote the manuscript. All authors provided critical feedback and helped to shape the research, analyses, and manuscript.

## Conflict of Interest

The authors declare that the research was conducted in the absence of any commercial or financial relationships that could be construed as a potential conflict of interest.

## Footnotes

1 www.uniprot.org

2 www.metaboanalyst.ca

3 https://www.uniprot.org/help/plants
