# Supplementary material for: Proteome-Wide Analysis of Heat-Stress in Pinus radiata Somatic Embryos Reveals a Combined Response of Sugar Metabolism and Translational Regulation Mechanisms
Source: Front Plant Sci. 2021 Apr 12;12:631239. doi: 10.3389/fpls.2021.631239 (PMC8072280; doi:10.3389/fpls.2021.631239)
Supplement: Supplementary file 1 [file Data_Sheet_1.docx]

Supplementary Material

# Supplementary Figures


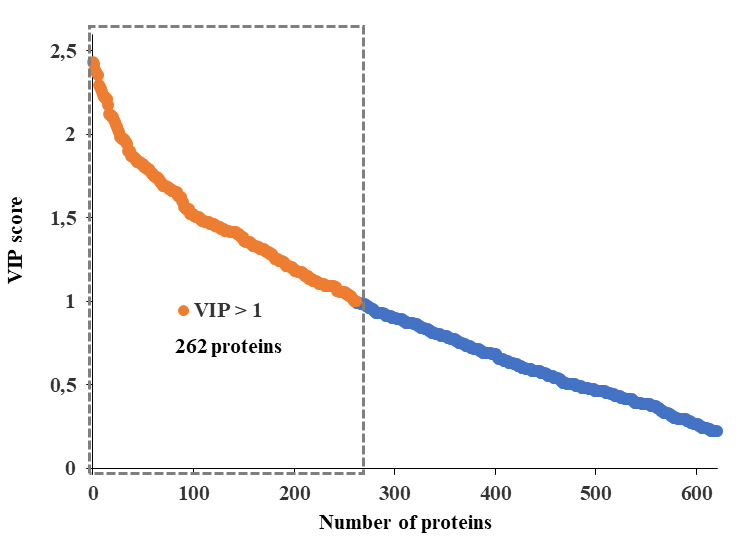


**Supplementary Figure 1.** Graphical representation of the results from the PLS-DA analysis showing that 262 proteins out of 758 presented VIP vales higher than 1. These proteins were considered the best classifiers and selected for further analyses.

**
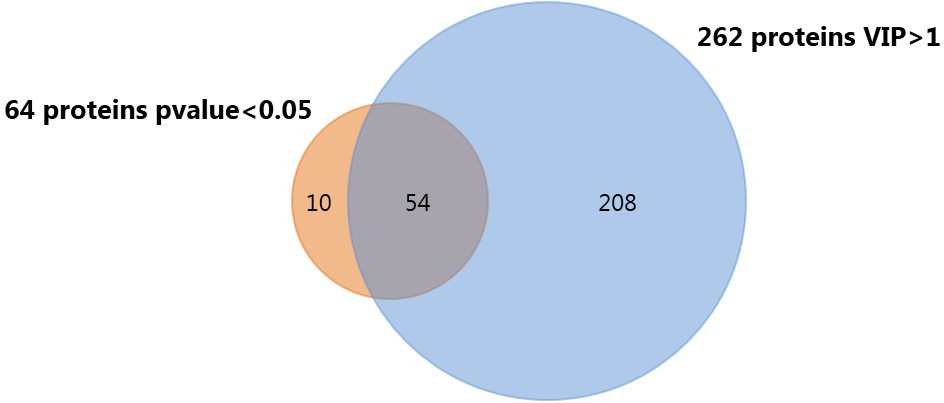
**

**Supplementary Figure 2.** Venn-diagram showing that 54 proteins were common between the 64 with *p* < 0.05 from the univariate analysis and the 262 resulted from the PLS-DA analysis with VIP > 1.


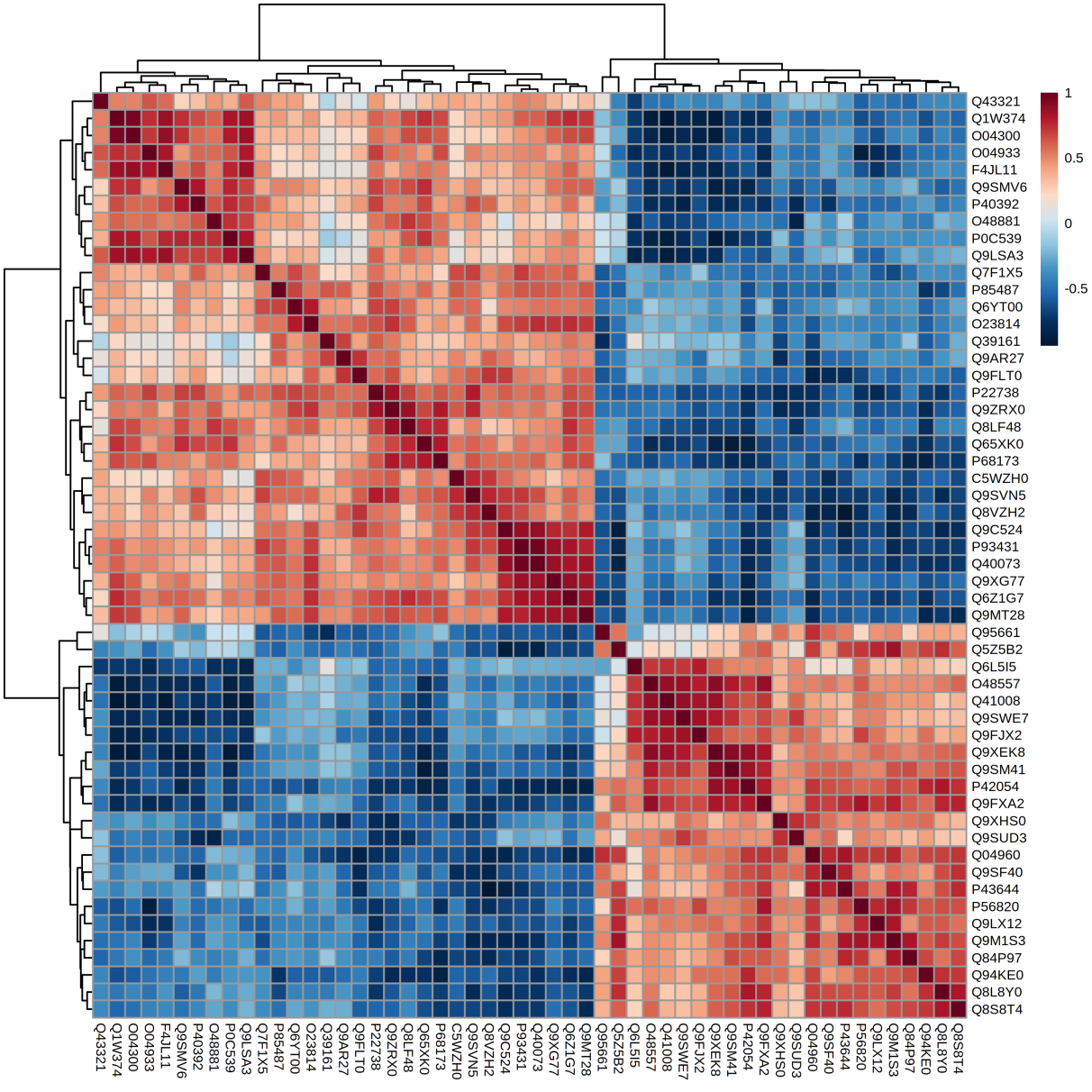


**Supplementary Figure 3.** Heatmap of the Spearman correlation coefficients of the 54 proteins selected by the combination of the Kruskal-wallis test (*p* < 0.05) and the PLS-DA analysis (VIP > 1) in somatic embryos of *P. radiata* originating from embryonal masses induced under high temperature conditions (Con1 = 23°C, 8 weeks, Cond2 = 40°C, 4 h, Cond3 = 60°C, 5 min). Red colour shows positive correlation whereas blue colour represents negative correlation among the selected proteins.

# Supplementary Tables

**Supplementary Table 1.** Excel file with raw data.

**Supplementary Table 2.** Proteins differentially accumulated in *P. radiata* somatic embryos obtained from embryonal masses induced under high temperature conditions (Con1 = 23°C, 8 weeks, Cond2 = 40°C, 4 h, Cond3 = 60°C, 5 min) according to the Kruskal-Wallis test (*p* < 0.05). Fold changes are presented as the ratio between one condition and the other.

|  |  | **Fold Change** | | | **Kruskal-Wallis** |
| --- | --- | --- | --- | --- | --- |
| **Description** | **Accession** | **Cond2/Cond1** | **Cond3/Cond1** | **Cond3/Cond2** | **p-value** |
| 60S ribosomal protein L3-2 | P22738 | 0,84 | 0,55 | 0,65 | 0,005 |
| Small heat shock protein, chloroplastic | Q95661 | 2,68 | 1,94 | 0,72 | 0,007 |
| Mitochondrial outer membrane protein porin 2 | Q6L5I5 | 0,86 | 1,37 | 1,60 | 0,010 |
| Outer plastidial membrane protein porin | P42054 | 1,10 | 1,58 | 1,43 | 0,010 |
| Multiple organellar RNA editing factor 8, chloroplastic/mitochondrial | Q9LKA5 | 1,34 | 1,06 | 0,79 | 0,010 |
| DnaJ protein homolog | Q04960 | 1,42 | 1,51 | 1,06 | 0,013 |
| Unknown protein 3 (Fragment) | P85487 | 0,66 | 0,47 | 0,71 | 0,013 |
| Actin-2 | P0C539 | 1,28 | 0,28 | 0,22 | 0,013 |
| Malate dehydrogenase 1, cytoplasmic | P93819 | 0,73 | 1,02 | 1,39 | 0,016 |
| Thiamine biosynthetic bifunctional enzyme BTH1, chloroplastic | O48881 | 1,03 | 0,71 | 0,69 | 0,016 |
| Ketol-acid reductoisomerase, chloroplastic | Q65XK0 | 0,75 | 0,39 | 0,52 | 0,016 |
| Translationally-controlled tumor protein homolog | Q9ZRX0 | 0,85 | 0,68 | 0,80 | 0,017 |
| Ribulose bisphosphate carboxylase/oxygenase activase,  chloroplastic | P93431 | 0,71 | 0,58 | 0,82 | 0,018 |
| Probable fructokinase-6, chloroplastic | Q9C524 | 0,70 | 0,65 | 0,93 | 0,018 |
| Ferredoxin--nitrite reductase, chloroplastic | Q39161 | 0,59 | 0,64 | 1,09 | 0,018 |
| 60S ribosomal protein L23 | Q9XEK8 | 0,98 | 1,37 | 1,40 | 0,018 |
| Peroxisomal fatty acid beta-oxidation multifunctional protein | Q8W1L6 | 0,60 | 0,88 | 1,46 | 0,019 |
| Pyruvate dehydrogenase E1 component subunit beta-1, mitochondrial | Q6Z1G7 | 0,89 | 0,69 | 0,78 | 0,020 |
| Probable transcription factor PosF21 | Q04088 | 0,61 | 0,51 | 0,83 | 0,020 |
| Ribulose bisphosphate carboxylase/oxygenase activase A, chloroplastic | Q40073 | 0,68 | 0,52 | 0,77 | 0,020 |
| Sugar transporter ESL1 | Q94KE0 | 1,19 | 1,77 | 1,49 | 0,021 |
| Casein kinase II subunit alpha-2 | Q9AR27 | 0,63 | 0,71 | 1,13 | 0,021 |
| Protein translation factor SUI1 homolog | Q9SM41 | 1,02 | 1,23 | 1,21 | 0,021 |
| Probable RNA-binding protein ARP1 | Q9M1S3 | 1,36 | 1,74 | 1,28 | 0,022 |
| Polyadenylate-binding protein 8 | Q9FXA2 | 1,24 | 1,57 | 1,27 | 0,022 |
| Aminopeptidase M1 | Q8VZH2 | 0,56 | 0,50 | 0,89 | 0,025 |
| Peptide methionine sulfoxide reductase A4, chloroplastic | Q336R9 | 0,71 | 1,20 | 1,69 | 0,025 |
| 60S ribosomal protein L30-3 | Q9LSA3 | 1,02 | 0,76 | 0,75 | 0,026 |
| Probable UDP-arabinopyranose mutase 1 | O04300 | 1,02 | 0,54 | 0,53 | 0,026 |
| Phosphomannomutase | Q1W374 | 0,95 | 0,65 | 0,68 | 0,026 |
| Inosine triphosphate pyrophosphatase | C5WZH0 | 0,84 | 0,80 | 0,95 | 0,026 |
| 60S ribosomal protein L17 | O48557 | 0,94 | 1,40 | 1,49 | 0,027 |
| Probable sucrose-phosphate synthase 2 | O04933 | 0,96 | 0,53 | 0,56 | 0,027 |
| 40S ribosomal protein S2-1 | Q8L8Y0 | 1,15 | 1,36 | 1,18 | 0,028 |
| 60S ribosomal protein L4-1 | Q9SF40 | 2,93 | 3,55 | 1,21 | 0,030 |
| Serine carboxypeptidase 3 | P21529 | 1,36 | 0,83 | 0,61 | 0,031 |
| DEAD-box ATP-dependent RNA helicase 7 | Q39189 | 0,84 | 1,99 | 2,37 | 0,031 |
| Eukaryotic translation initiation factor 3 subunit D | P56820 | 1,20 | 1,46 | 1,22 | 0,032 |
| Proteasome subunit alpha type-6 | Q9XG77 | 0,88 | 0,81 | 0,91 | 0,032 |
| Protein argonaute 1D | Q5Z5B2 | 1,67 | 1,66 | 0,99 | 0,032 |
| Threonine synthase, chloroplastic | Q9MT28 | 0,87 | 0,77 | 0,89 | 0,033 |
| Probable inositol 3-phosphate synthase isozyme 3 | Q9LX12 | 1,97 | 3,20 | 1,62 | 0,034 |
| Proteasome subunit alpha type-7-A | Q6YT00 | 0,86 | 0,79 | 0,92 | 0,036 |
| Ras-related protein RIC1 | P40392 | 0,59 | 0,36 | 0,60 | 0,037 |
| V-type proton ATPase subunit E | Q9SWE7 | 1,02 | 1,25 | 1,22 | 0,038 |
| Chaperone protein dnaJ A6 | Q0JB88 | 1,25 | 1,07 | 0,86 | 0,038 |
| 3-ketoacyl-CoA thiolase 1, peroxisomal | Q8LF48 | 0,98 | 0,72 | 0,74 | 0,038 |
| Importin subunit alpha-2 | F4JL11 | 1,05 | 0,76 | 0,72 | 0,039 |
| 60S ribosomal protein L13a | O65055 | 0,74 | 0,95 | 1,28 | 0,039 |
| Acetyl-coenzyme A carboxylase carboxyl transferase subunit  alpha, chloroplastic | Q41008 | 1,06 | 1,46 | 1,37 | 0,039 |
| 40S ribosomal protein S12 | Q9XHS0 | 1,06 | 1,37 | 1,29 | 0,039 |
| Enolase | Q43321 | 0,93 | 0,04 | 0,05 | 0,039 |
| Probable phospholipid hydroperoxide glutathione peroxidase | O23814 | 0,69 | 0,62 | 0,89 | 0,040 |
| Cysteine proteinase inhibitor A | Q10992 | 0,80 | 1,06 | 1,32 | 0,040 |
| Mitochondrial outer membrane protein porin 5 | Q84P97 | 1,09 | 1,28 | 1,17 | 0,042 |
| Aminomethyltransferase, mitochondrial | P54260 | 0,70 | 1,00 | 1,42 | 0,046 |
| 60S ribosomal protein L26-2 | Q9FJX2 | 1,02 | 1,36 | 1,33 | 0,046 |
| DnaJ protein homolog ANJ1 | P43644 | 1,29 | 1,34 | 1,04 | 0,046 |
| Ribonuclease TUDOR 2 | Q9FLT0 | 0,67 | 0,69 | 1,03 | 0,047 |
| Fumarate hydratase 1, mitochondrial | P93033 | 0,80 | 0,80 | 0,99 | 0,047 |
| UDP-glucuronic acid decarboxylase 4 | Q8S8T4 | 1,11 | 1,32 | 1,19 | 0,048 |
| 60S ribosomal protein L12 | O50003 | 0,89 | 1,06 | 1,19 | 0,048 |
| Cytochrome c oxidase subunit 6b-3 | Q9SUD3 | 1,19 | 1,47 | 1,24 | 0,048 |
| Uncharacterized protein At2g24330 | Q9ZQ34 | 1,83 | 1,38 | 0,75 | 0,049 |
